# The Assessment of Methyl Methanesulfonate Absorption by Amphipods from the Environment Using Lux-Biosensors

**DOI:** 10.3390/bios14090427

**Published:** 2024-09-05

**Authors:** Uliana S. Novoyatlova, Anna A. Kudryavtseva, Sergey V. Bazhenov, Anna A. Utkina, Vadim V. Fomin, Shamil A. Nevmyanov, Bagila S. Zhoshibekova, Maria A. Fedyaeva, Mikhail Y. Kolobov, Ilya V. Manukhov

**Affiliations:** 1 Moscow Center for Advanced Studies, Moscow 123592, Russiabazhenov1994@gmail.com (S.V.B.);; 2 A.N. Severtsov Institute of Ecology and Evolution of the RAS, Moscow 119071, Russia; 3 Youth Educational Expeditions, Dolgoprudny 141701, Russia; 4 Department of Biology, Kazakh National Women’s Teacher Training University, Almaty 050000, Kazakhstan; 5 Faculty of Biology, Lomonosov Moscow State University, Moscow 119991, Russia; 6 Laboratory of Microbiology, BIOTECH University, Moscow 125080, Russia

**Keywords:** biosensor, alkylation, environmental control, methyl methanesulfonate, amphipod, absorption

## Abstract

The ability of aquatic mesofauna representatives involved in trophic chains to sorb and accumulate toxicants is important for understanding the functioning of aquatic ecosystems and for fishing industry. This study investigated the capacity of marine amphipod *Gammarus oceanicus* and freshwater amphipods *Eulimnogammarus vittatus* and *Gammarus lacustris* to absorb the DNA-alkylating agent methyl methanesulfonate (MMS). The presence of alkylating agents in the environment and in the tissues of the amphipods was determined using whole-cell lux-biosensor *Escherichia coli* MG1655 pAlkA-lux, in which the *luxCDABE* genes from *Photorhabdus luminescens*, enabling the luminescence of the cell culture, are controlled by the P*_alkA_* promoter of DNA glycosylase. It was shown that within one day of incubation in water containing MMS at a concentration above 10 μM, the amphipods absorbed the toxicant and their tissues produce more alkylation damage to biosensor cells than the surrounding water. Concentrations of MMS above 1 mM in the environment caused the death of the amphipods before the toxicant could be significantly concentrated in their tissues. The sensitivity and the capacity to absorb MMS were found to be approximately the same for the marine amphipod *G. oceanicus* and the freshwater amphipods *E. vittatus* and *G. lacustris*.

## 1. Introduction

Amphipods have long been used as test subjects for assessing the degree of water pollution. The first works concerning the use of amphipods as vital test organisms came about in the second half of the 20th century, such as [1,2,3]. Since that time, toxicological studies using amphipods continue. In the work [4], the influence of species, sex, and biochemical dimorphism on the sensitivity to various toxicants was investigated. The impact on various systems of amphipods, such as reproductive function, mass gain, the ability to metabolically degrade toxicants, or the overall mortality of test organisms, is used to refine the sensory capabilities of amphipods in assessing environmental pollution and in toxicological studies of new chemicals. Methods for assessing the accumulation of toxicants in the bodies of various crustaceans, including amphipods, were developed and actively used. Mostly such works concern the accumulation of heavy metals and metalloids [5,6]. In the studies [5,7], the accumulation of heavy metals in specific organs and subcellular distribution was investigated.

In the work by [8], the cumulative toxic effect of organic compounds such as dichloro-diphenyl-trichloroethane and polychlorinated biphenyls on amphipods was demonstrated. For several organic and inorganic compounds, accumulation in river sediments and benthic organisms has been demonstrated [9]. For instance, *Gammarus pulex* has shown the ability to accumulate some perfluorinated compounds but not certain pharmaceuticals and illicit drugs. A bioaccumulation model of organic compounds in *G. pulex* has been developed, using a range of organic compounds that are primarily used as pesticides [10]. Overall, the ability of amphipods to bioaccumulate organic compounds is still poorly represented in the literature, and the development of new research methods remains relevant.

Methyl methanesulfonate (MMS), used in this study, is an organic compound widely used as a research chemical in the field of genetics and molecular biology and as a solvent catalyst in polymerization, alkylation, and esterification reactions [11,12]. MMS toxicity is based on the transfer of methyl groups to DNA, which leads to the formation of mutagenic lesions. This property makes it a valuable tool for inducing mutations in laboratory settings, allowing researchers to study gene function and DNA repair mechanisms. MMS is reasonably anticipated to be a human carcinogen [11,13]. Exposure to methyl methanesulfonate mostly appears to be limited to laboratory research personnel as the chemical is not used in industry [14].

There are several methods for toxicity assessment and bioaccumulation determination. There are physical methods for the determination of chemical compounds in the tissues of aquatic species, such as liquid chromatography–tandem mass spectrometry [9], the measurement of radioactivity from ^14^C [10], and others. There are methods that measure not the chemical, but the toxic effect; one of them is the Ames test, which evaluates the rate of mutagenesis [15,16]. Another example is SOS-chromotest, which allows the determination of DNA-damaging agents [17]. Currently, biotesting methods using bacterial lux-biosensors, i.e., bacterial cells capable of bioluminescence [18], are widely used to determine environmental contamination by toxic substances. Natural luminescent microorganisms are typically used to determine overall (or integral) toxicity, which is assessed by measuring bioluminescence quenching, meaning that when the viability or metabolism of a cell is impaired, its luminosity falls down [19,20]. Lux-biosensors based on genetically engineered strains with the *lux* genes under stress-inducible promoters are more sensitive than naturally luminescent bacteria and additionally possess specificity to the toxicant class [21,22]. It was shown that the results obtained with stress-inducible biosensors correlate with those obtained with the Ames test [23]. The use of whole-cell lux-biosensors has its own advantages and limitations. In general, physico-chemical methods for the determination of chemicals in the medium and in the tissues tend to be more sensitive [5,6]. However, lux-biosensors can evaluate the bioavailability and toxic effect on a living cell, which may be more important in toxicity studies of new compounds and their metabolites and for assessing environmental risks [18].

In the present work, we used lux-biosensor *Escherichia coli* pAlkA-lux [24], which is specific to the alkylation of DNA nitrogenous bases, to test the ability of amphipods to bioconcentrate the DNA-alkylating agent methyl methanesulfonate (MMS). Three species of amphipods were used: the marine *G. oceanicus* and the freshwater *E. vittatus* and *G. lacustris*.

## 2. Materials and Methods

### 2.1. Test Organisms

In this work, several amphipod species were used. Marine amphipods *G. oceanicus* (Segerstråle, 1947) were collected in the lower littoral zones, among fucus and ascophyllum areas between boulders near Olenyevsky Island in the Chernorechenskaya Bay of the Kandalaksha Gulf in the White Sea, Russia (66.523651° N, 33.116682° E). Freshwater amphipods E. *vittatus* (Dybowsky, 1874), ranging in size from 6 to 16 mm, were collected in the coastal waters of Lake Baikal near Olkhon Island, Khuzhir rural settlement, Russia (53.194886° N, 107.330970° E). Freshwater *Gammarus lacustris* (G.O. Sars, 1863) were collected in Kabanye Lake (Novosibirsk region, Western Siberia, Russia).

Pasteurized seawater (collected from the *G. oceanicus* collection site) and filtered freshwater (collected from the *E. vittatus* collection site) were used for incubation of marine and freshwater organisms, respectively. For *G. lacustris,* the artificial water was prepared as follows: reverse osmosis water with the addition of a complex of essential salts for natural water bodies, including sodium, magnesium, and calcium (Kent Marine RO Right, Franklin, WI, USA). The total mineralization level of the artificial water was 0.1 ppm. Groups of a few similarly sized amphipods (up to 20) were placed in containers with aerated water. In the experiment, one container did not contain MMS, while the others contained concentrations ranging from 1 μM to 10 mM. The amphipods were incubated in the water for 24 h without feeding at a temperature of approximately 8–12 °C. The temperature was chosen considering the water temperature in the native environment of the amphipods. The incubation time was chosen experimentally so that the effect of bioaccumulation may be observed and was less than the decomposition time of MMS in these conditions. The next day, the amphipods were removed from the water in which they had been incubated and rinsed in water without MMS. Each individual was placed in a microtube and homogenized with the addition of 30 μL of distilled water. After homogenization, the samples were centrifuged at 10,000 rpm to separate the liquid and solid fractions, and 20 μL of the supernatant was used for further analysis. 

### 2.2. Bacterial Strains

In the work, the whole-cell lux-biosensors detecting DNA alkylation and general toxicity were used; the strain and plasmids characteristics are listed in the Table 1. 

### 2.3. Culture Medium and Growth Conditions

*E. coli* cell cultures were prepared by being cultivated overnight at 37 °C with 200 rpm aeration in 25 mL glass culture tubes containing 5 mL of LB media supplemented with 100 μg/mL ampicillin. These cultures were then diluted 1:100 in LB and grown at 37 °C until they reached an OD_600_ of 0.1–0.2, indicating the early logarithmic phase. The resulting cultures were utilized for further experiments and luminescence measurements.

### 2.4. Measurement of Bioluminescence

Bioluminescence intensities were measured in 200 μL portions of cell culture using capeless microtubes with a Biotox-7BM (BioPhysTech, Dolgoprudny, Russia). Biosensor cells were placed into the 200 μL subcultures in separate tubes, and 20 μL of the tested compound (water or supernatant from homogenized amphipod tissue) was added. The cells were incubated without shaking at room temperature, with repeated direct measurements of total bioluminescence. Luminescence values were expressed in relative light units (RLUs).

The induction coefficients were calculated for all bioluminescence values. To determine the luminescence induction coefficient of biosensor cells, the following equation was used:

$$K_{ind}(t,x)=\frac{Lum(t,x)}{Lum(t,contr)}$$

where K_ind_ (t, x) is the calculated luminescence induction coefficient of biosensor cells in sample x at time point t, and where Lum(t, contr) is the luminescence in the control sample of the same biosensor cells with the addition of the non-toxic solution.

As the concentration of the toxicant in the environment increases, the biosensor cells begin to sense specific damage and the luminescence increases in dose-dependent manner; high concentrations of the toxicant lead to significant disruptions in cell function and the luminescence decreases. In order not to confuse high concentrations with low ones, we used a biosensor with constitutive synthesis of luciferase, the luminescence of which will decrease if the toxicity of the sample is high enough for vital functions of *E. coli* cells.

## 3. Results

**Figure 1:**
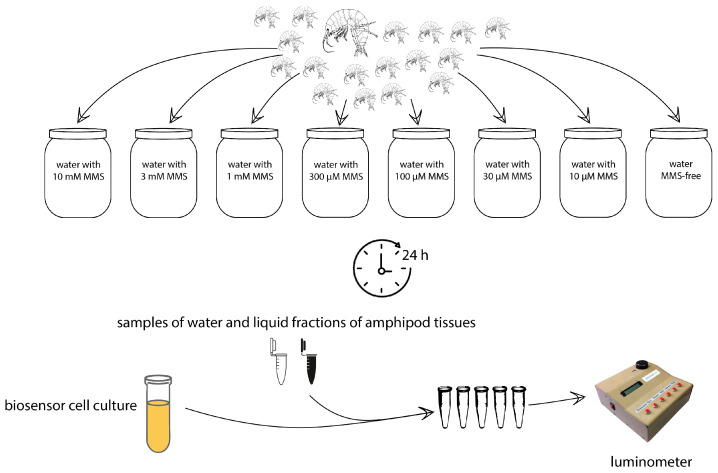
Scheme of the experiment. Amphipods are divided into groups and incubated in water with various concentrations of MMS or without it. After 24 h, the incubation alkylation ability of amphipod’s tissues and water from the flasks/jars was tested using the whole-cell lux-biosensors.

**Figure 2:**
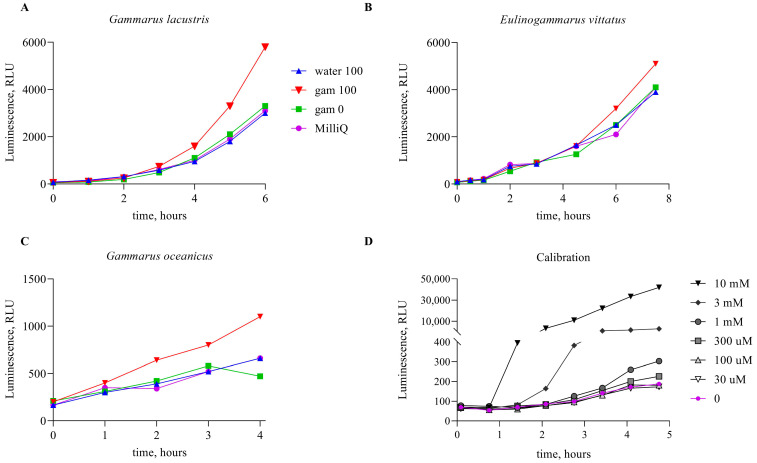
(**A**–**C**)—Luminescent signal of *E. coli* MG1655 pAlkA-lux biosensor upon the addition (1/10 V) of water and the liquid fraction of homogenized amphipods incubated in this water. Curve “water 100”—MMS-supplemented (100 µM) water sampled after incubation of amphipods in it. “gam 100”—amphipods after incubation in MMS-supplemented (100 µM) water; “gam 0”—amphipods after incubation in MMS-free water, “K-”—MMS-free water sampled after incubation of amphipods (negative control). As test objects, the following amphipods were used: (**A**)—*G. lacustris*, (**B**)—*E. vittatus* (**C**)—*G. oceanicus*, *s*. (**D**)—Luminescent signal of *E. coli* MG1655 pAlkA-lux biosensor upon the addition (1/10 V) of water with MMS added in various concentrations (calibration samples).

To study the ability of marine amphipods to accumulate MMS, *G. oceanicus*, *E. vittatus*, and *G. lacustris* gathered from nature were used. The collected organisms were divided into the groups and incubated during 24 h in water with MMS at concentrations ranging from 1 µM to 10 mM or without any supplementations (control group). The scheme of the experiment is given in Figure 1. It appeared that MMS in concentrations between 1 and 10 mM in the water is fatal to amphipods within a few hours. After 24 h incubation, amphipods were isolated, rinsed with fresh water, and homogenized. The liquid fraction of homogenized tissues was used to determine the content of alkylating agents using bacterial biosensor cells *E. coli* MG1655 pAlkA-lux. Figure 2 shows the kinetics of luminescence activation in the cells upon the addition of the liquid fraction from homogenized amphipods incubated in presence of 100 µM MMS. Also, graphs show biosensor signals upon the addition of the following samples: MMS-free water, in which control group of amphipods was incubated; liquid fractions from homogenized amphipods from the control group; and the water with 100 µM MMS, sampled after 24 h incubation of amphipods.

As can be seen from Figure 2, the induction of biosensor luminescence occurs upon the addition of samples from amphipods which were incubated in the MMS-supplemented water. In contrast, the sample of the surrounding water does not induce bioluminescence, nor does the sample of amphipods that were kept in MMS-free water. The data presented in the Figure 2 were obtained mostly in expeditions, so it was not possible to keep the same incubation temperature in each case. This affected the time of activation and the character of the induction curves, so they slightly differ for each experiment. 

It should be noted that when water samples containing 100 µM MMS are added to a culture of biosensor cells, the final concentration of MMS is 10 µM (1/10 V addition), which is below the threshold concentration that causes the activation of the lux-biosensor according to [27]. Therefore, it is not surprising that in our experiments, water with 100 µM MMS, in which gammarids were incubated, did not cause a biosensor response. The effect of the activation of the luminescence of *E. coli* MG1655 pAlkA-lux cells by gammarid tissues, shown in Figure 2, can be explained by the accumulation of MMS in concentrations above 100 µM.

**Figure 3:**
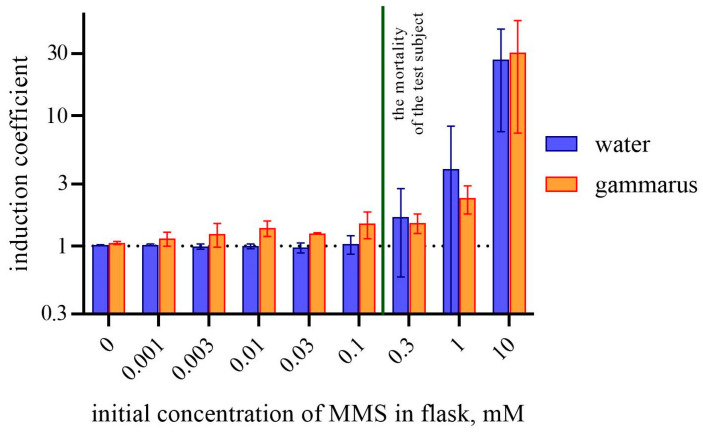
Comparison of the induction coefficient of *E. coli* MG1655 pAlkA-lux after 5 h of incubation following the addition of the liquid fraction of homogenized amphipods, incubated in MMS-containing water, or the water, in which amphipods were incubated. Abscissa values correspond to MMS concentration in water in the beginning of the experiment. All the values from three replicates of experiments from three gammarid species are combined. The dashed line show the value of 1 for induction coefficient (the value indicating the absence of biosensor induction).

The nature of the induction and the low values of induction coefficients indicate that there is no significant difference between all the species of amphipods used, so the data for all species were combined. Induction coefficients were calculated for all the experiments and combined in Figure 3 (average for all amphipods). In Figure 3, the induction coefficients are shown for amphipod tissues and MMS-supplemented water, sampled after the 24 h incubation of amphipods.

In the Figure 3, a significant difference between bars representing the biosensor culture with water and gammarid tissues can be observed for MMS concentrations in the medium from 0.003 to 0.1 mM—water does not induce the biosensor cells, while gammarid tissues produce detectable alkylation damage to the bacterial cells. At concentrations above 0.1 mM, the induction coefficient for the water sample increased, which is consistent with the biosensor calibration (Figure 2D). At the same time, when MMS in water causes the mortality of amphipods, the induction of biosensor cells by gammarid tissues no more exceeds the signal from the water. It may be connected to the death of the test organisms and their incapability to absorb any toxicants. MMS at concentrations above a few mM has a general toxic effect on bacteria too and can lead to a decrease in luminescence. We monitored this effect on biosensor cells *E. coli* MG1655 pDlac with *lux* genes located under the constitutive promoter (see Supplementary Materials). It was shown that the concentrations reducing the level of bacterial luminescence are higher than 1 mM, which corresponds to 10 mM MMS in the added water sample (1/10 V). This means that the majority of probes analyzed in this study did not affect the vital functions of bacterial cells.

## 4. Discussion

The data presented in this work allow us to assert that amphipods living in water containing DNA-alkylating substances can absorb such substances in their bodies. According to the report of the US Department of Health and Human Services, MMS should be hydrolyzed in a moist environment, and it is not expected to bioconcentrate in aquatic organisms [28].

Our findings indicate the opposite. We did not investigate the ability of amphipods to metabolize MMS, but our data indicate that, at least during the experiment, MMS degradation was not noticeable or the products retained their alkylating capacity. The ability to accumulate alkylating agents is demonstrated by the increased alkylating activity of *G. oceanicus*, *E. vittatus,* and *G. lacustris* tissue samples compared to the MMS-containing medium in which they were incubated. However, we really cannot say based on our data about the accumulation of MMS itself in gammarid tissues, since stress-specific biosensors allow the detecting of only biological effects rather than exact chemicals. We can only say for sure about the appearance of some compounds in gammarid tissues that have an alkylating potential and are capable of damaging DNA. In Figure 3, the detection of alkylating substances in tissues of gammaridss incubated in an aqueous medium with MMS at concentrations from 3 to 100 μM was demonstrated. These concentrations of MMS in the medium itself are not detected by bacterial biosensors.

The task of assessing the content of alkylating agents in the environment is highly relevant due to the spread of mutagenic compounds, which arise from some household waste [29], the insufficient purification of chemical production products, and other aspects of human activity. In some research [30,31], the toxic effects of detergents and cleaning agents on the environment, animals, and humans was described, including health issues. Various methods (DNA comet assay, Ames test, Allium test) were used to investigate the mutagenicity of detergents in the article [29]. It was shown that some of the detergents possess mutagenic properties. Additionally, there are many other possible sources of alkylating pollutants, including less obvious ones such as rocket propellant pollution and nitrate pollution. The compound unsymmetrical dimethylhydrazine (UDMH), being a component of rocket fuel, has highly toxic properties. There are several works proving that UDMH spills cause long-term consequences for human health and the environment [32,33,34], and the main toxic effect of UDMH is believed to be DNA alkylation [24] and the formation of reactive oxygen species that damage DNA [35]. The effects of nitrate pollution on the environment and organisms are widely known [36,37]. The mechanisms of nitrate toxicity continue to be investigated, and there are reports of the alkylating potential of nitrate sources from poultry manure [38]. The results of our work will be applicable to assessing the risks associated with the ability of aquatic mesofauna representatives involved in trophic chains to sorb and accumulate toxicants. Such studies are important for the implementation of environmental monitoring, understanding the functioning of aquatic ecosystems and fishing industry product safety assurance.

## 5. Conclusions

In this work, we showed that alkylating compounds can be absorbed by aquatic organisms from the environment. Potentially, the technology we describe could constitute a method to detect sublethal concentrations of alkylating compounds in organisms. This may make it possible to prevent serious environmental pollution from genotoxic compounds at an early stage.

## Figures and Tables

**Table 1:** Bacterial strain and plasmid used in the study.

Name		Description	Source
strain	*E. coli* MG1655	F- ilvG rfb-50 rph 1	(VKPM, Russia)
plasmids	pAlkA-lux	pDEW201 [25] vector-based plasmid pAlkA-lux, where P*_alkA_* promoter from *E. coli* is transcriptionally fused with *luxCDABE* genes from *P. luminescens*. Ampicillin resistance (Ap^r^)	[24]
	pDlac	Plasmid based on pDEW201 with the insertion of a P*_lac_* promoter	[26]

## Data Availability

The data are contained within the article.

## Supplementary Materials

The following supporting information can be downloaded at: https://www.mdpi.com/article/10.3390/bios14090427/s1, Figure S1: Effect of addition of MMS-supplemented water on the luminescence of the *E. coli* MG1655 pDlac cells.
