# Supplementary material for: The Assessment of Methyl Methanesulfonate Absorption by Amphipods from the Environment Using Lux-Biosensors
Source: Biosensors (Basel). 2024 Sep 5;14(9):427. doi: 10.3390/bios14090427 (PMC11430740; doi:10.3390/bios14090427)
Supplement: Supplementary file 1 [file biosensors-14-00427-s001.zip › biosensors-3154342-supplementary.pdf]

Assessment of methyl methanesulfonate absorption by amphipods from the environment using *lux*-biosensors

Novoyatlova U.S. [1,2], Kudryavtseva A.A. [1], Bazhenov S.V. [1], Utkina A.A. [1], Fomin V.V. [1], Nevmyanov Sh.A. [3], Zhoshibekova B.S. [4], Fedyaeva M.A. [5], Kolobov M.Yu. [2,5] and Manukhov I.V. [1,6,*]

Supplementary Material

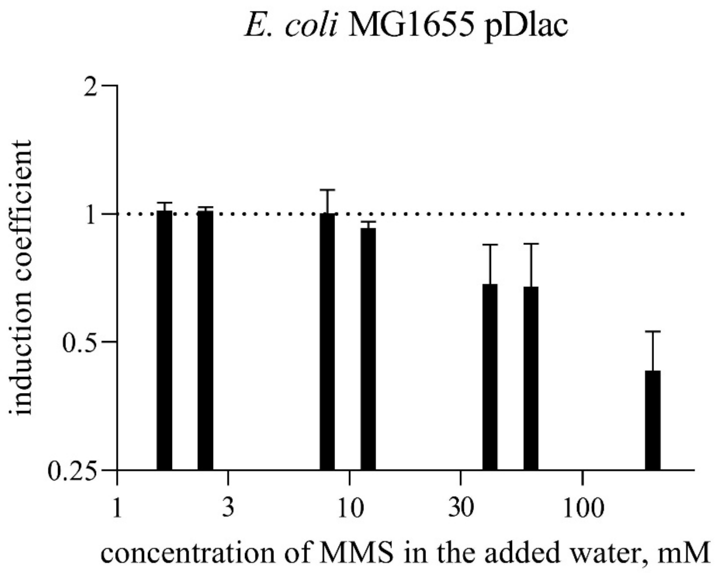

Figure S1. Effect of addition of MMS-supplemented water on the luminescence of the *E. coli* MG1655 pDlac cells. MMS-supplemented water has been added to the cell culture in a ratio of 1/10 (V/V).
